# Solution Synthesis of Cubic Spinel Mn–Ni–Cu–O Thermistor Powder

**DOI:** 10.3390/ma14061389

**Published:** 2021-03-12

**Authors:** Duc Thang Le, Heongkyu Ju

**Affiliations:** Department of Physics, Gachon University, Seongnam 13120, Korea; ducthang36@skku.edu

**Keywords:** co-precipitation, cubic spinel, nanoparticle, nickel manganite, sintering, thermistor

## Abstract

Toward the development of NTCR thermistors, nanocrystalline Mn–Ni–Cu–O powder was synthesized from a mixed chloride aqueous solution by a simple co-precipitation method.The introduction of an oxidizing agent (H_2_O_2_) into the solution led to the partial oxidation of Mn^2+^ ions into Mn^3+^ ions, which enabled the collected powder to be well crystallized at 650 °C. Such a low calcining temperature resulted in fine particles with a mean size of 60 nm, which significantly promoted densification of the resulting ceramics. As a result, a dense and homogenous microstructure with a relative density up to 97.2% was achieved for pellets sintered at 1100 °C. Furthermore, these sintered ceramics exhibited a room temperature resistivity (*ρ*_25_) of 67 Ω·cmand a thermistor constant (*B*_25/85_) of 2843 K, which make them suitable for use in industrial thermistors. In addition, electrical stability was greatly improved when the ceramics were prepared by a new two-step sintering method. The results suggest that the co-precipitation route with the introduction of H_2_O_2_ is suitable for the fabrication of cubic spinel thermistor nanopowders.

## 1. Introduction

Semiconducting nickel manganite ceramics are widely used in industrial thermistors owing to their high negative temperature coefficient of resistance (NTCR), environmental friendliness, and cost effectiveness [1,2,3,4]. These materials have a spinel-type structure with the general formula AB_2_O_4_(A: tetrahedral sites; B: octahedral sites) and conduct electricity through electron hopping between the Mn^3+^ and Mn^4+^ states at the B sites (instead of traditional electronic conduction) [3,5,6]. Particularly, their resistivity decreases exponentially with temperature, which can be described by Arrhenius equation [4,5] *ρ*(*T*) = *ρ*_0_exp(*Ea/k_B_T*), where *ρ*_0_ is the resistivity at infinite temperature, *T* is the absolute temperature, *k_B_* is Boltzmann constant, and *E_a_* is the activation energy for the hopping process.

The NTCR characteristic ofthermistors is often determined based on three basic parameters: room temperature (*RT*) resistivity *ρ*_25_, (Ω·cm), *B* constant (K), and aging coefficient (Δ*R*/*R*, %). These parameters significantly depend onthe material composition. Hence, various transition metal ions have been added into nickel manganites in different ways to improve the sensing performance [1,4,6]. Among the transition elements, Cu is the most effective for enhancing the conductivity of nickel manganitesdue to the insertion of monovalent copper (Cu^+^) ions into the A sites [7,8,9], which leads to the displacement of Ni^2+^ cations from the A sites to the B sites of the spinel structure. Therefore, ceramics with Mn–Ni–Cu–O composition have become one of the most important thermistor materials.

NTCR thermistors are typically produced by sintering powder precursors at elevated temperatures, which consolidates the powder particles into dense monoliths with tailored microstructures and properties. Sintering is a grain growth-induced densification process enabled by atomic diffusion (solid-state reactions) under the driving force of heat energy. To achieve thermistors with high density and minimal porosity, high sintering temperatures and long dwell times are generally employed in the conventional sintering process [10,11,12]. However, at such high temperatures, the spinel structure becomes unstable and decomposes into rock salt (NiO) and Mn-rich spinel phases [10,11,13,14], resulting in the deterioration of the electrical performance. It has been reported that the use of fine powders allows the synthesis of thermistors at relative low sintering temperatures because of the high surface free energy of the composed particles, and thus prevents phase decomposition [15]. In practice, high-performance thermistors are generally obtained from nanopowders having a single cubic spinel structure [16].

NTCR nickel manganite-based powders can be synthesized by two main approaches: solid-state routes and solution routes [17]. Although the solid-state synthesis method is simple and cost effective, it results in compositional inhomogeneity and produces large particles. In contrast, solution processes yield powders with better compositional homogeneity and fine particles; however, controlling the powder stoichiometry during the preparation is complicated [18,19]. As mentioned above, a complete spinel formation is necessary which requires a heat treatment process, known as “calcining” or “pre-sintering.”Nickel manganite-based powders crystallize at various firing temperatures; however, the stable cubic spinel phase generally forms at a temperature of 750 °C and above, regardless of the preparation methods [20,21]. Such high temperatures increase the particle size of the resulting oxides. In most of practical works, the size of the calcined particles generally ranges from several hundreds of nanometers to a few micrometers [22,23].

Much effort has been devoted to reducing the particle size by different ways to improve the sintering activity of the synthesized powders. For example, prolonged ball milling has been performed as an additional mechanical activation step after calcination to obtainnano-sized particles [16,24]. The authors noted that ball milling not only decreased the particle size, but also changed the crystal structure of the oxide compounds. Fang et al. synthesized fine powders from oxalate compounds by the solid-state coordination reaction method, which is an intermediate between the solid-state and solution techniques [17,25,26]. In this route, raw precursor powders were dry milled at *RT* to obtain a solid-state coordination compound, which was then calcined at elevated temperatures to obtain the powders with the desired stoichiometry. As a result, oxide powder with a particle size of 200–300 nm was achieved after calcination. Although these techniques successfully produced small-sized powders, thereby enhancing the sinterability of bulks and thick films, the mechanical milling process renders the control of particle morphology/uniformity, size distribution, purity, and reproducibility somewhat difficult.

**Figure 1:**
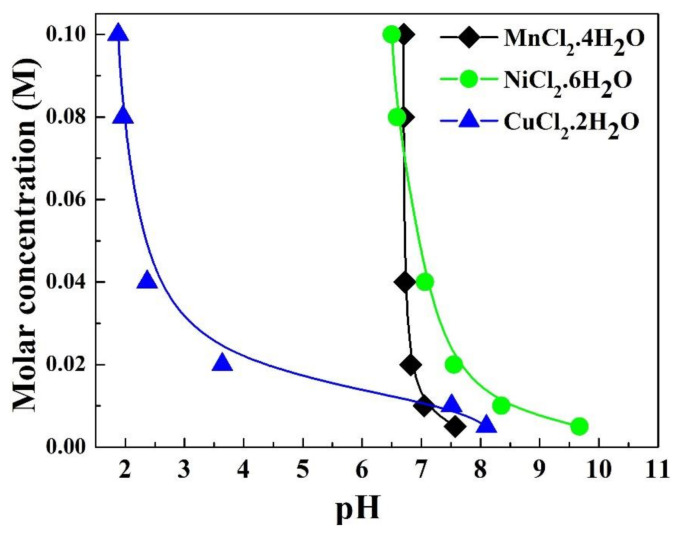
Concentration vs. pH plot showing liquid-precipitate transformation of Mn^2+^, Ni^2+^, and Cu^2+^ ions at 60 °C. The diagram was plotted based on the data of experiment series, presented in ref [28].

We considered that if stable cubic spinel symmetry can be achieved at a relatively low temperature, particle growth at high temperatures can be significantly suppressed, and thus, smaller particles can be obtained. In our previous work [27], we synthesized hausmannite (Mn_3_O_4_) thin films from chloride solutions by liquid flow deposition (LFD). We then prepared thin films of (Ni, Cu, Mn)_3_O_4_ [28] cubic spinel-type oxides with excellent electrical properties by the same method. The pH was controlled in the range of 6.65–6.85 to deposit the thin films. However, precipitates can easily form in the solution at pH over 7.00, which reveals the possibility of preparing manganite powders. The formation of precipitates at a high pH is associated with liquid–solid phase transformation, as shown in Figure 1, and is discussed in detail elsewhere [28]. In addition, the LFD films crystallized with a cubic spinel structure at a firing temperature as low as 300 °C, and this single phase was stable at temperatures above 400 °C [29].

Based on this background, we expected that cubic spinel powders could be synthesized by a similar solution route. Accordingly, we adopt aco-precipitation method [30] to synthesize Cu_0.3_Ni_0.66_Mn_2.04_O_4_powder in this work. The targeted powder was produced from a chloride solution by adding H_2_O_2_. The changes in the morphology and phase of the powder with calcination temperature were examined. Furthermore, the electrical properties of the resulting ceramics fabricated by both conventional single step sintering and new two-step sintering methods were demonstrated.

## 2. Experimental Procedure

### 2.1. Preparation of Powder and Ceramics

High purity (99.99%) MnCl_2_·4H_2_O, NiCl_2_·6H_2_O, and CuCl_2_·6H_2_O (Sigma-Aldrich, St. Louis, MO, USA) were used as raw materials. All reagents were accurately weighed to obtain a Cu:Ni:Mn molar ratio of 0.3:0.66:2.04. The mixture was dissolved in distilled water to form a 0.1 M solution in a flat-bottom flask. Then, NH_4_Cl (Daejung, Siheung-si, Gyeonggy-do, Korea) was added to the mixture and stirred for 1 h at 60 °C using a magnetic stirrer to obtain a liquid solution. Subsequently, ammonia (NH_3_·H_2_O, Daejung, Siheung-si, Gyeonggy-do, Korea) was added dropwise with a pipette to adjust the pH to approximately 7.05. When the solution pH stabilized, an appropriate amount of H_2_O_2_ (5 mM) was slowly added under continuous stirring for another 1 h. The pH was then adjusted to 9.5 and the solution was held at this pH for an additional 30 min. The resulting precipitates were collected without any washing or filtering, and then slowly dried in a vacuum oven (Daihan, WON-32, Wonju-si, Gangwon-do, Korea) by heating to 120 °C at a heating rate of 10 °C/h. The obtained powder was calcined in air for 2 h at different temperatures of 300, 400, and 650 °C at a heating rate of 5 °C/min. To prepare bulk ceramics, the oxide mixture calcined at 650 °C was ground, blended with 3 vol% polyvinyl alcohol (Daejung, Siheung-si, Gyeonggy-do, Korea), and cold pressed into discs with a diameter of 12 mm and thickness of 5 mm at 200 MPa. The green compacts were covered with alumina crucibles and sintered in a conventional furnace (Alarge, QSH-1400M, Shanghai, China).

### 2.2. Characterizations

#### 2.2.1. Powders

The morphology of the powders was observed by field-emission scanning electron microscopy (FE-SEM, 6700F, JEOL, Tokyo, Japan) and transmission electron microscopy (TEM, JEM 2100, JEOL, Tokyo, Japan). X-ray diffraction (XRD, RAD III, Rigaku, Tokyo, Japan) was used to analyze the crystalline structure and was then confirmed by Fourier-transform infrared spectroscopy (FT/IR-6700, JASCO, Easton, MD, USA). The chemical composition of powders was investigated by inductively coupled plasma optical emission spectroscopy (5110 ICP-OES, Agilent, Santa Clara, CA, USA). For ICP-OES analysis, the Borate fusion process was applied to prepare the solutions. Accordingly, each powder was first added into a flux mixture of LiBO_2_ and LiBr with a ratio of 1:10 and heated in a Fluxer (Katanax X-600, Metuchen, NJ, USA) at 1050 °C for 7 min. The melting mixture was then poured in a dilute acid solution (HNO_3_, 10%) to obtain the final solution.

#### 2.2.2. Ceramics

The bulk density (*δ_s_*) of the sintered ceramics was measured by Archimedes method, and the relative density (*δ*_rl_) was calculated by *δ*_rl_ = *δ*_s_/*δ*_x_(%), where *δ*_x_ is the X-ray density [31]. The oxidation states of manganese and copper were examined by an X-ray photoelectron spectroscopy (XPS) system (ESCA 3000, VG Microtech, London, UK). For electrical resistance measurement, the sintered samples were lapped to 1 mm thickness and electroded by applying silver paste on both the surfaces using a screen printer. After the paste air dried, the samples were cured at 750 °C in air for 15 min at 5 °C/min of the heating/cooling rates. The samples were then kept in a holder in a silicon oil bath, and the temperature was measured using a digital thermometer. A digital multimeter (Fluke 45, 8808A, Everett, WA, USA) was used to measure the electrical resistance in the temperature range of 293–391 K. The *B* constant was calculated as *B*_25/85_ = 1778 × (ln*R*_25_ − ln*R*_85_), where *R*_25_ and *R*_85_ are the bulk specific resistances (Ω) at 25 °C (298 K) and 85 °C (358 K), respectively. The aging coefficient was determined from the relative change in resistance Δ*R*/*R* = (*R* − *R*_0_)/*R*_0_ × 100%, where *R*_0_and *R* are the resistances at *RT* (298 K) before and after aging at 150 °C (in air) for 500 h. The *ρ*_25_, *B*_25/85_, and Δ*R*/*R*values are the averages of five measured samples.

## 3. Results and Discussion

### 3.1. Characterization of Powder

**Figure 2:**
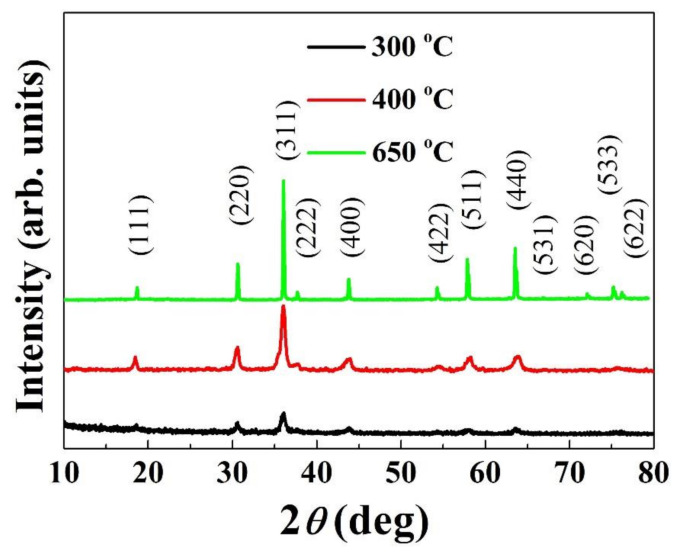
XRD patterns of oxide powder calcined at different temperatures.

The XRD patterns of the powder calcined at different temperatures are shown in Figure 2. As shown in Figure 2, distinct peaks appeared at 300 °C corresponding to the (111), (220), (311), (222), (400), (422), (511), (531), (620), (533), and (622) crystal planes, which imply that the powder was crystallized with a cubic spinel structure (JCPDS Card No. 84–0542). The appearance of broad XRD peaks implies a small crystallite size and/or a low degree of crystallinity [32]. With an increase in calcination temperature, the peaks became sharper with higher intensity and fewer humps. This indicates the promotion of crystallization and crystallite growth with increasing temperature. Notably, a well-crystallized Cu_0.3_Ni_0.66_Mn_2.04_O_4_ spinel is found at 650 °C, implying that the cubic spinel formation was complete at this temperature. Otherwise, regardless of the calcination temperature, all the powders exhibited the same cubic spinel structure without any secondary phase, although withdifferent degrees of crystallinity. In other words, no structural change or dissociation occurred in the studied temperature range, which would be an evidence for the phase stability. This finding is different from the results of the previous studies [20,21] on nickel manganite-based compounds, where a phase decomposition (into NiMnO_3_ and α-Mn_2_O_3_) was found in the temperature range of 400–750 °C.

**Figure 3:**
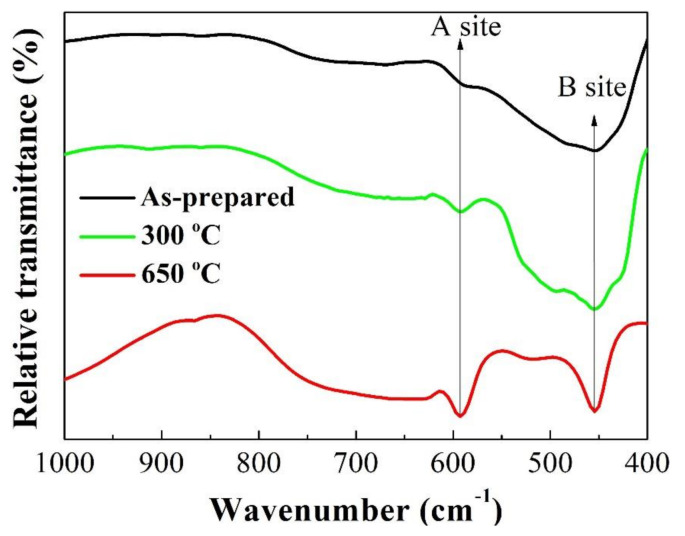
FT–IR spectra of as-prepared and calcined powders.

The FT–IR spectra of the as-synthesized and calcined powders are presented in Figure 3. The FT–IR spectra were recorded in the frequency range of 400–1000 cm^−1^ to confirm the spinel structure of the synthesized powder. Generally, the formation of a spinel structure associates with two absorption bands between 400 cm^−1^ and 600 cm^−1^ [30]. The higher band correspondsto the stretching vibration of metal–oxygen bonds at the A site, and the lower band is attributed to the stretching modes of the B site [30,32,33]. In this case, two frequency bands appeared around 589–592 cm^−1^ and 452–455 cm^−1^ (Figure 3), similar to those reported for cubic spinel Fe_0.48_Ni_0.3_Mn_1.32_Co_0.9_O_4_ powders synthesized by the sol-gel route [32]. These peaks became stronger and more distinct with increasing temperature, which indicates (i) the crystallization occurs during the calcination and (ii) the crystallinity degree increases with calcining temperature. These are in good agreement with the XRD results shown in Figure 2.

**Figure 4:**
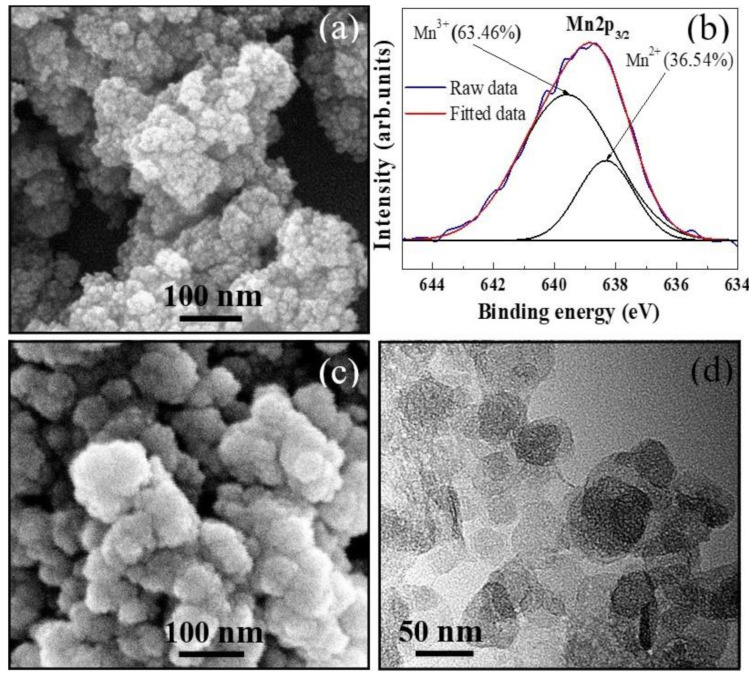
(**a**) FE-SEM image and (**b**) XPS Mn2p_3/2_ spectra of as-prepared powder. (**c**) FE-SEM and (**d**) TEM images of powder calcined at 650 °C.

To determine the change in microstructure during the calcination process, FE-SEM observation was performed on the as-prepared and calcined powders; the results are shown in Figure 4a,c. To minimize the surface free energy, particles commonly tend to aggregate into dense clusters with a large size [16]. Such agglomerations were observed in both the as-prepared and calcined powders; however, single particles were also present in these powders. In particular, Figure 4a reveals the presence of spherical particles of <20 nm diameter, which indicates that very fine powders can be synthesized under the experimental conditions of the proposed technique. The powder calcined at 650 °C contained larger particles with a size of up to 80 nm, as shown in Figure 4c. The mean size of these oxide particles was approximately 60 nm, as confirmed by a TEM observation shown in Figure 4d. Obviously, particle growth occurred during calcination nevertheless, the particle size of the calcined powder (Figure 4c,d) was much smaller than those synthesized by other techniques [15,16,17,18,22,23,24,25,26,32]. We attribute the narrow size range of the oxide particles to the low calcination temperature (650 °C). In addition, the particles exhibited high uniformity (Figure 4d) with a uniform shape and a narrow size range, which can be attributed to the synthesis process that does not involve any mechanical impact.

Inductively coupled plasma optical emission spectroscopy (ICP-OES) was performed to determine the chemical composition of the as-prepared and calcined (650 °C) powders; the results are presented in Table 1. As can be seen, the ratios of the three constituent elements (Cu:Ni:Mn) in both the powders were similar to the Cu:Ni:Mn ratio in the initial solution. These results indicate that at a high pH (~9.5), the constituent elements (Cu, Ni, and Mn) completely transformed into a solid phase, which can be understood by examining the liquid–precipitate transformation characteristic of these elements (Figure 1). It can thus be reasonably concluded that the chemical composition of the powder depends only on the raw materials. Because of this reason, high purity (99.99%) salt chlorides were used in this work.

### 3.2. Crystal Structure and Microstructure of Sintered Ceramics

To investigate the sintering behavior and electrical properties, the powder calcined at 650 °C was pressed into pellets, which were then sintered in a conventional furnace. In general, to achieve a high electrical performance, thermistors must have a single cubic spinel phase and a dense body (relative density > 95%). In this work, to prevent the decomposition of the NiO phase, which generally occurs at high temperatures, we sintered the pellets in air by the conventional single step (SS) sintering at a relatively low temperature of 1100 °C (the samplesare denoted as S1). In addition, the new two-step (TS) sintering method has been demonstrated as a feasible approach to produce cubic spinel nickel manganite-based ceramics [34]. Accordingly, to provide more information about the properties of ceramics derived from the synthesized powder, the samples were also fabricated by TS sintering (denoted as S2), under the conditions listed in Table 2.

**Figure 5:**
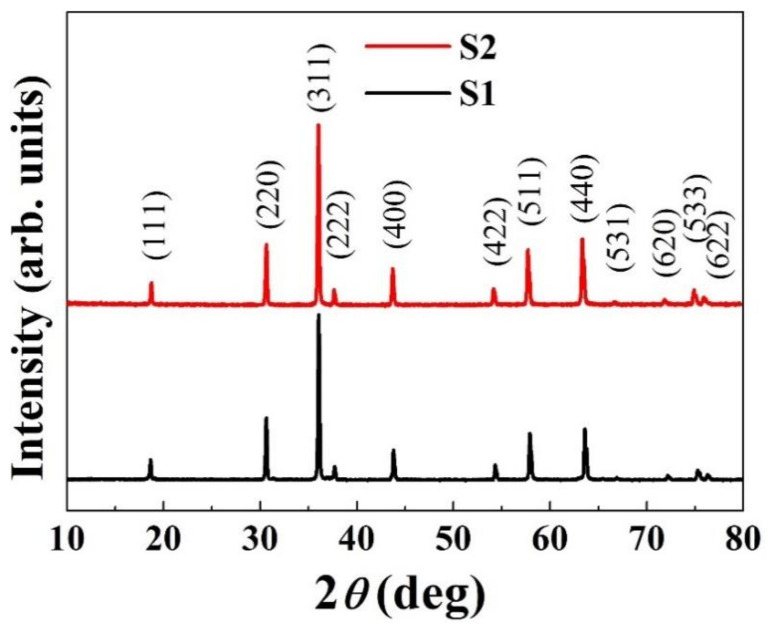
XRD patterns of sintered ceramics.

The XRD patterns of S1 and S2 are shown in Figure 5. As expected, both the sintered pellets exhibited a cubic spinel structure. Obviously, no phase transformation or segregation occurred during SS sintering at 1100 °C. This is in good agreement with the findings of previous studies on NiMn_2_O_4_ ceramics [10,11]. For the case of S2, it is commonly accepted that a high temperature (1200 °C) was applied in the first step to obtain a critical density, which may cause a NiO phase decomposition. However, in the second step of the TS sintering, a cubic spinel phase was formed due to the phase re-transformation (from NiO) at a lower temperature (900 °C) [35]. A similar result of cubic spinel phase was demonstrated by Ma et al. for NiMn_2_O_4_ ceramics via TS sintering [34].

**Figure 6:**
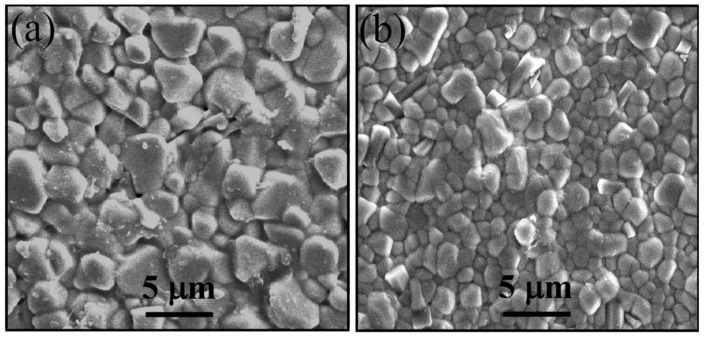
Surface FE-SEM micrographs of (**a**) S1 and (**b**) S2 ceramics.

Figure 6 shows the FE-SEM images of the fracture surfaces of the fabricated ceramics. It is seen that both the specimens exhibited dense and homogenous microstructures with well-grown grains. A dense microstructure of sintered pellets is a prerequisite for ensuring good electrical properties and reproducibility of thermistors. The average grain size of S1 was 3.4 μm (Figure 6a), whereas that of S2 was 1.2 μm (Figure 6b). Clearly, the grain size of S2 is nearly three times smaller than that of S1, which confirms that ceramics with fine grains can be fabricated by the TS sintering. Moreover, the relative densities (*δ*_rl_) of S1 and S2 were measured as 97.2% and 97.5%, respectively, which indicate the high densification of the ceramics derived from the synthesized powder. It is noteworthy that the sintering temperature of 1100 °C, which led to the high *δ*_rl_ (>97%) of S1, is lower than those required to prepare ceramics with the same density level from solid-state powders [9,14,16,36,37]. In fact, a *δ*_rl_ of approximately 97.5% was obtained without any change in phase composition when the specimens were sintered for longer than 6.5 h. The excellent sinterability of the resulting ceramics can be attributed to the small size and uniformity of the calcined powder (Figure 4c,d), which endows the green compacts with a *δ*_rl_ as high as 63.4% under a pressure of 200 MPa. Further, the large free surface energy of the fine particles [15,16] promotes the contact and reaction between them during sintering, resulting in high-density products.

### 3.3. Electrical Properties of Sintered Ceramics

**Figure 7:**
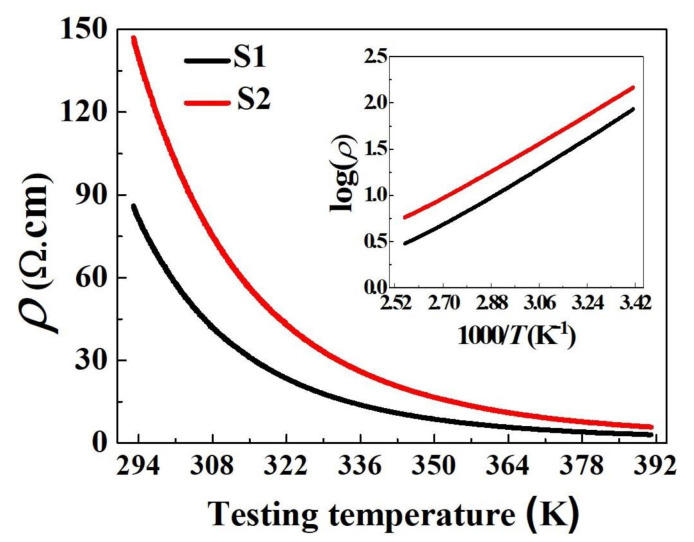
Resistivity−temperature (*ρ*−*T*) curve of sintered ceramics. The insert shows a plot of log(*ρ*) versus (1000/*T*).

To examine the electrical properties, the electrical resistivity-temperature dependence was measured in the temperature range of 293–391 K, and the results are presented in Figure 7. It is observed that the resistivity (*ρ*) progressively decreases with increasing temperature (*T*), indicating a typical NTCR characteristic. In addition, the inset of Figure 7 shows a nearly linear relationship between the log(*ρ*) and reciprocal absolute temperature 1000/*T* for both fabricated ceramics, which can be attributed to the polaron hopping mechanism, whereby the *ρ*–*T* dependence is explained by Arrhenius equation [4,5,34,35,36,37,38]. In general, electrical conduction in spinel manganites occur through hopping mechanism between the Mn^3+^ and Mn^4+^ states at the B sites. In the case of Cu-doped nickel manganite, a solid-state charge transfer redox system exists that oxidizes Mn^3+^ to Mn^4+^ and reduces Cu^2+^ to Cu^+^ [39,40,41]. Further, the monovalent copper occupies the A sites, while bivalent copper occupies both A and B sites of the spinel [8,9]. The presence of Cu^+^ increases the amount of cations in the A sublattices, which results in the displacement of some Ni^2+^ cations from the A sites to the B sites. This displacement produces Mn^4+^ at the B sites for charge compensation, consequently increasing the concentration of Mn^4+^ ions. The larger amount of Mn^4+^ ions, more Mn^3+^/Mn^4+^ couples were available for electron hopping at the B sites, which is responsible for the electrical conductivity of the fabricated ceramics shown in Figure 7.

It has been reported that the optimal electrical conductivity of spinel ceramics wasattained only when the Mn^3+^/Mn^+4^ ratio isunity [42]. In our case, this ratio is found to be 0.955 and 0.973 for S1 and S2, respectively (Table 3). These values are closed to unity, which demonstrate that the nearly maximum electrical performance was achieved for both ceramic samples.

The measured electrical properties of the sintered ceramics are summarized in Table 4. It is observed that both the ceramics are highly conductive, and their technical parameters are desirable for NTCR thermistors [3]. However, the specific *ρ*_25_ and *B*_25/85_ of S1 (67Ω·cm and 2843 K) are slightly lower than those of S2 (116 Ω·cm and 3012 K). The differences in *ρ*_25_ and *B*_25/85_ imply that the sintering method affects the electrical properties even though the ceramics have the same chemical composition. In this study, because S1 and S2 have not only the same chemical composition, but also a very similar crystal symmetry (Figure 5), we attribute the difference in the resistivity of the two ceramics to the grain size. The finer grains in Figure 6b indicate the presence of a higher number of grain boundaries in S2. The larger the number of grain boundaries, the larger is the boundary resistance, and thus, the lower is the conductivity.

**Figure 8:**
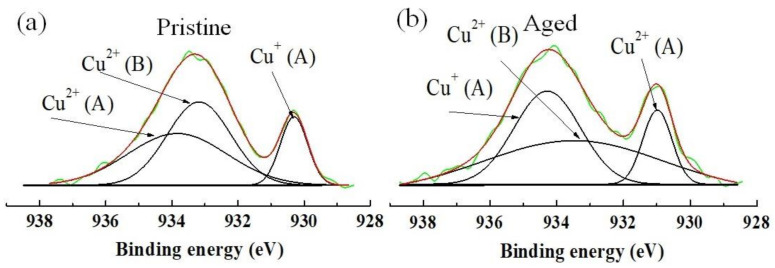
XPS spectra in the Cu2p_3/2_ core level of S1 ceramic examined before (**a**) and after (**b**) aging at 150 °C in air for 500 h.

In practice, under thermal constraint, most NTCR thermistors undergo an increase in electrical resistance over time, so called resistance drift, which is also known as aging [6,35]. The aging behavior is presented by aging coefficient (Δ*R*/*R*) and it reflects the electrical stability of the materials. The Δ*R*/*R* should be as small as possibleto maintain the reproducibility of the thermistor parameters such as *ρ*_25_ and *B*_25/85_. In this work, the Δ*R*/*R* was 5.65% for S1 ceramics (Table 4), which is comparable to that of the Cu_0.3_Ni_0.66_Mn_2.04_O_4_bulks derived from mixed oxalate powder [43] but much lower than those of conventional Cu-doped nickel manganites [6,44]. The underlying mechanism of resistance driftin Cu-containing nickel manganites generally involves cationic oxidation and cationic migration from the A sites to the B sites in the spinel structure [45,46]. Particularly, the Cu^+^ ions at the A sites oxidize to Cu^2+^ during aging (heating in an air atmosphere), which results in the migration of Cu ions from the A sites to the B sites. This ion migration decreases the Mn^4+^ content, and therefore leads to an increase in resistance [44,47]. In our case, the relative low aging coefficient of S1 is possibly because of an increase in the concentration of Cu^2+^ ion and a decrease in the concentration of Cu^+^ ion in the pristine ceramics due to the relatively low sintering temperature [43]. Such a low Cu^+^ concentration and a high Cu^2+^ concentration suppress Cu^+^ – Cu^2+^ oxidation during aging, and thus, restrict the ion migration. The migration of Cu cations is confirmed quantitatively by XPS Cu2p_3/2_analysis, as shown in Figure 8. According to the previous literatures [48], the order of binding energies (BE) in the Cu2p_3/2_ region is Cu^+^ (A) < Cu^2+^ (B) < Cu^2+^ (A). Accordingly, the detail Cu2p_3/2_characteristics of S1 in pristine and aged states are listed in Table 5. It can be clearly seen that neither the Cu^+^ (A) nor Cu^2+^ (B) changed much after the aging process. Moreover, as compared to S1, the S2 ceramics show a lower Δ*R*/*R*(3.32%). Clearly, the smaller specific aging coefficient value of S2 indicates that more stable ceramics can be fabricated by the TS sintering. This behavior can be attributed to the dense and fine-grained microstructure in Figure 6b, which retards the adsorption of oxygen, and thus, the formation of cationic vacancies in the spinel lattices. The fine-grained microstructure was also reported to remarkably contribute tothe high thermal stability in the case of Ni_0.7_Mn_2.3_O_4_ [35] and (LaMn_0.5_Co_0.5_O_3_)*_x_*(Ni_0.66_Mn_2.34_O_4_)_1–*x*_ [49] thermistors.

It should be noted that the high electrical performance of the resulting ceramics can be an evidence for the success of the co-precipitation method used for the fabrication of Cu_0.3_Ni_0.66_Mn_2.04_O_4_ thermistor powder. Therefore, we believe that this method can be extended to fabricate other NTCR powders with (M, Ni, Mn)_3_O_4_ (M = Co, Fe, Zn, Al, Mg, etc.,) composition, which will be addressed in our next studies.

### 3.4. Formation Mechanism of Spinel Powder

The important finding of this work is that oxide powder with a pure cubic spinel phase can be obtained via calcination at 650 °C. Such a relatively low calcining temperature leads to the formation of ultrafine particles, which in turn improves the sinterability, and thus, the electrical performance of the resulting ceramics. The co-precipitation method employed in this study offers two main advantages: (i) homogeneous composition, and (ii) coexistence of Mn^2+^ and Mn^3+^ ions in the collected precipitates. It has been reported that the homogeneous mixing of starting components reduces the crystallization temperature of the resulting powders [50]. Moreover, Mn^3+^ ions were created by the chemical oxidation of Mn^2+^ to Mn^3+^ ions in the solution, driven by H_2_O_2_, consequently, the mass fraction of Mn^3+^ was 63.46% as determined by in XPS analysis (Figure 4b). Because such a Mn^3+^ fraction already existed in the as-prepared powder, less (or even no more) Mn^2+^→Mn^3+^ oxidation reactions are required (to provide Mn^3+^ ions for a spinel formation) in the solid phase, which possibly lowers the thermal budget for crystallization. Therefore, we attribute the coexistence and chemical homogenous dispersion of Mn^2+^ and Mn^3+^ to the major factors responsible for the low critical crystallization temperature (300 °C) and high crystallinity at 650 °C. Otherwise, because the Mn^3+^ ions originated from Mn^2+^ (the same source), the diffusion distance may be shorter than that in normal cases in which Mn^2+^ and Mn^3+^ ions come from different sources; this could be another reason for the low-temperature formation of the spinel structure. Further, we suggest that because the Mn^2+^→ Mn^3+^ oxidation reactions are suppressed by Mn^3+^ cations pre-existence, NiMnO_3_ and α-Mn_2_O_3_ phases were not formed during calcination [20,21,51]; consequently, a stable cubic spinel phase was obtained.

The preparation method used in this work is similar to the LFD technique used for the synthesis of Mn_3_O_4_-based thin films [27,28,29]. The difference between the two methods is that LFD thin films are formed via heterogeneous (ion-by-ion) nucleation, while the powders prepared in this study are formed in solution by a homogenous (cluster-by-cluster) process. We therefore propose the following scheme for the formation of a spinel powder in this study:

NH_4_Cl → NH^4+^ + Cl^−^ (1)

NH^4+^ + OH^−^ ↔ NH_3_·H_2_O (2)

M^2+^ (M = Mn, Ni, and Cu) cations combine with NH_3_ (aq.) to form metal amine complexes (M(NH_3_)*_n_*^2+^) via the following reaction [52]:

M^2+^ + *n*NH_3_ ↔ Mn(NH_3_)_n_^2+^  (*n* = 1–4) (3)

At pH > 7, OH^−^ groups are available in the solution and combine with the metal amine complexes as follows:

M(NH_3_)_n_^2+^ + OH^−^ → [M(NH_3_)_n_](OH)_2_  (*n* = 1–4) (4)

Once the oxidizing agent (H_2_O_2_) is added into the system, a part of M^2+^ ions (Mn^2+^, but may include Ni^2+^) oxidize to form M^3+^:

3[Mn(NH_3_)_n_](OH)_2_ + H_2_O_2_ → [Mn^2+^,Mn^3+^]_3_O_4_ + 4H_2_O + 3*n*NH_3_ (5)

Eventually, upon heat treatment, the spinel phase is formed:

(6) $${\left[{\mathrm{Mn}}^{2+},{\mathrm{Mn}}^{3+}\right]}_{3}O_{4}\overset{\mathrm{Heat}\;\mathrm{treatment}}{\to }[({\mathrm{Mn}}^{2+}){\left({\mathrm{Mn}}^{3+}\right)}_{2}]O_{4}$$

## 4. Conclusions

Nanocrystalline Cu_0.3_Ni_0.66_Mn_2.04_O_4_ powder was successfully synthesized from a mixed chloride aqueous solution by a simple co-precipitation method. The addition of H_2_O_2_ induced the partial oxidation of Mn^2+^ ions into Mn^3+^ ions, which resulted in the onset of crystallization at 300 °C. The single cubic spinel phase was stable with calcining temperature and exhibited improved crystallinity at 650 °C. The calcined powder was composed of uniform particles with a mean size of 60 nm, which promoted the densification of the pellets during sintering. As a result, the specimens sintered at a relatively low temperature of 1100 °C exhibited a high relative density of 97.2%, while retaining a pure cubic spinel structure. These sintered ceramics exhibited average *ρ*_25_ and *B*_25/85_ of 67 Ω·cm and 2843 K, respectively, which are desirable for NTCR thermistors. Moreover, the ceramics showed greater electrical stability when prepared by a new two-step sintering method.

## Figures and Tables

**Table 1:** Chemical composition of the fabricated powder.

Elements	Chemical Composition		
	Solution	As-Repared	Calcined at 650 °C
Cu	0.30	0.33	0.32
Ni	0.66	0.67	0.63
Mn	2.04	2.00	2.05

**Table 2:** Sintering conditions used in this study.

Sintering Methods	*T*_1_ (°C)	Soaking Time (min)	*T*_2_(°C)	Soaking Time (min)
SS ^1^			1100	240
TS ^2^	1200	10	900	240

^1^ SS: single step. ^2^ TS: two step.

**Table 3:** XPS Mn2p_3/2_ analysis of sintered ceramics.

Samples	Peak Intensity (%)			Mn^3+^/Mn^4+^ Ratio
	Mn^2+^	Mn^3+^	Mn^4+^	
S1	22.12	38.05	39.83	0.955
S2	18.45	40.23	41.32	0.973

**Table 4:** Electrical properties of sintered ceramics.

Samples	*ρ*_25_ (Ω·cm)	*B*_25/85_ (K)	Δ*R*/*R* (%)
S1	67 ± 3	2843 ± 2	5.65 ± 0.2
S2	116 ± 3	3012 ± 2	3.32 ± 0.2

**Table 5:** Detailed characteristics of the XPS Cu2p_3/2_ spectra in Figure 8.

Samples	Binding Energy (eV)			Peak Intensity (Area %)		
	Cu^+^ (A)	Cu^2+^ (B)	Cu^2+^(A)	Cu^+^ (A)	Cu^2+^ (B)	Cu^2+^ (A)
Pristine	930.2	933.2	933.9	16.29	41.56	42.15
Aged	930.1	933.2	934.1	14.68	39.54	45.78

## Data Availability

The data presented in this study are available on request from the corresponding author. The data are not publicly available due to privacy.
